# Evolutionary game model of migraine based on the human brain hypersensitivity

**DOI:** 10.3389/fneur.2023.1123978

**Published:** 2023-03-29

**Authors:** Dong-Gyun Han

**Affiliations:** Dr. Han's Neurology Clinic, Daejeon, Republic of Korea

**Keywords:** migraine, reciprocal altruism, evolutionary game model, hypersensitivity, evolutionarily stable state

## Abstract

Based on all studies published up to 2020, the prevalence of migraine worldwide is approximately 14%, although it varies regionally. Despite being one of the most disabling diseases, migraine still exists through natural selection and is prevalent today. This raises the question of what evolutionary advantages have led to the survival of migraine. The ultimate answer to this question should be found in evolution; however, there is no clear explanation yet. Notably, all the genes that cause migraine make the sensory organs and cortex of the migraine sufferer hypersensitive. In a state of hypersensitivity, the brain could recognize external threats easily. Game theory is a useful tool for explaining evolution in terms of genes. Just as the Hawk–Dove game, which has two strategies (aggressive and passive) and four fitness values, an evolutionary game between a migraineur and a non-migraineur, which shows two phenotypes (more sensitive and less sensitive) and four fitness values, can be played if a migraineur quickly recognizes a predator and informs a non-migraineur of its appearance and the non-migraineur later helps the migraineur escape from danger. This study aimed to explore the evolutionary mechanics of migraine that can be modeled. Furthermore, it tried to define why the human brain's hypersensitivity is a prerequisite for developing this evolutionary game model.

## 1. Introduction

There are two explanations for life's phenomena. One is a proximate cause, explaining “how”, and the other is an ultimate cause, explaining “why”. The ultimate cause for “why” is an evolutionary explanation (1). The proximate causes of migraine that have been discovered to date are, at the physiological level, the neuronal hypersensitivity of sensory organs or cortices resulting from mutations in the genes that control glutamate or gamma-aminobutyric acid activity at the biochemical level (2). At the neuroanatomical level, migraines are caused by the activation and sensitization of the trigeminocervical complex (TCC) interacting with the amygdala-hypothalamus (3, 4), dysregulation of sensory information through the thalamus (5), abnormal pain control by the periaqueductal gray-rostral ventromedial medulla (PAG-RVM) pathway (6, 7), and activation of the locus coeruleus (LC) leading to a hypersensitive state of the TCC or sensory cortices (8). At the level of evolutionary psychology, migraine is a kind of tonic immobility or a primitive reflex. Sensory hypersensitivity and over-reactivity to stimuli revive this primitive defense reflex retained in humans. Migraine shows an intense pulsing or throbbing pain in the head or headache, in addition to systemic reactions of a parasympathetic surge (loss of muscle tone, decreased reactivity, hypotension, and bradycardia) (9, 10). These systemic reactions are like tonic immobility (11–14).

The enormous number of proximate causes of migraine that have been discovered to date at each of the above levels are disconnected, disparate, and often controversial with each other, so they confuse us in our search for the basic pathophysiological mechanism of migraine (15, 16). Nonetheless, at present, by wide consensus, migraine is considered a primary brain disorder; migraineurs' brains are hypersensitive to stimuli and show altered sensory processing (17–20). In the interictal period, migraineurs show hypersensitivity to sensory stimuli and abnormal processing of sensory information, characterized by a lack of habituation of evoked and event-related potentials (17, 21, 22). As the neurophysiological mechanism of migraine, it has been proposed that hypofunctioning serotonergic projections to the thalamus and cortex cause functional disconnection of the thalamus, leading to thalamocortical dysrhythmia and reduced cortical habituation and hypersensitivity (17, 21). Finally, the interictal cortical hypersensitivity to sensory stimuli demands an exaggerated amount of energy, thereby initiating the chemical cascade that leads to migraine attacks.

Despite recent remarkable advances in migraine pathophysiology and therapy, an evolutionary mechanism has not been produced. The evolutionary cause for migraine will now be discussed. As Theodosius Dobzhansky once said, “nothing in biology makes sense except in the light of evolution.” The structures and functions of living things can be understood from an evolutionary perspective. The present structures and functions of living things have contributed to their survival and reproduction in the past. Under the logic of evolution, we can also determine the ultimate cause of our species-specific disease or migraine. Migraine is experienced by approximately 14% of the world's population. According to the 2020 Global Burden of Disease Study (23), it is one of the most disabling diseases. As a highly prevalent, often severely painful, and frequently disabling disease, migraine should have ceased to exist through natural selection; however, it is prevalent to date. This raises the question of what evolutionary advantages have led to migraine survival (24). Hence, this study attempts to explore the evolutionary mechanics of migraine through reciprocal altruism and game theory.

## 2. Methods and results

In evolutionary biology, reciprocal altruism is a behavior in which an individual temporarily sacrifices oneself to help another individual, keeping in mind the possibility that the other will later help in a similar way (25). For reciprocal altruism between migraineurs and non-migraineurs to work, three conditions must be met. First, individuals must interact more than once so that the opportunity to be repaid can arise. Second, individuals must be able to reliably recognize other individuals. Third, individuals must remember the past behavior of those with whom they interact.

**Figure 1:**
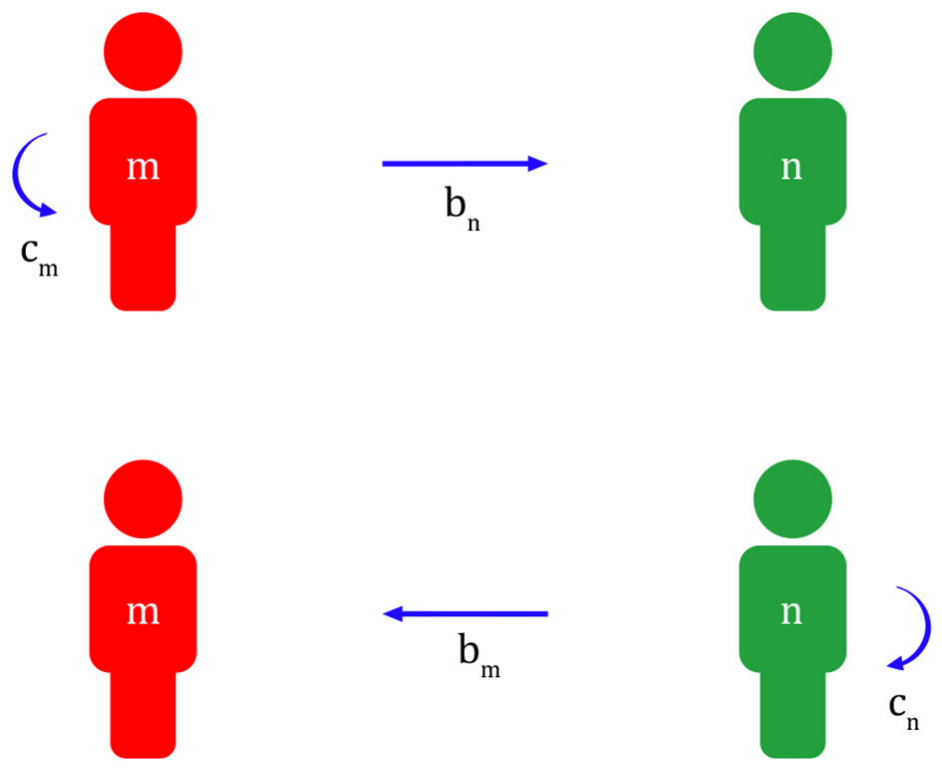
Reciprocal altruism between a migraineur and a non-migraineur (m, migraineur; n, non-migraineur; c_m_, cost of migraineur; b_m_, benefit of migraineur; c_n_, cost of non-migraineur; b_n_, benefit of non-migraineur; b_m_ - c_m_ >0, b_n_ - c_n_ >0).

Applying this to migraine, a migraineur with a more sensitive nervous system can quickly recognize a predator and inform their colleague, or a non-migraineur with a less sensitive nervous system, to its appearance at risk of being exposed. Later, in return, the non-migraineur will help the migraineur escape danger if a predator appears during a migraine attack (Figure 1).

A migraineur has the added cost c_m_ of maintaining a highly sensitive nervous system and informing a non-migraineur of the appearance of a predator at risk of being exposed. In this case, a non-migraineur with a less sensitive nervous system gets help b_n_ from a migraineur. Subsequently, the non-migraineur has the cost c_n_ of helping the migraineur get out of danger if a predator appears during a migraine attack. In this case, the migraineur gets b_m_ from the non-migraineur. However, to give and receive help from each other, b_m_-c_m_ > 0, b_n_-c_n_ > 0 must always be established.

To date, migraine remains highly prevalent worldwide. There are no regional or racial exceptions to migraine. Thus, the fact that migraine genes have continued to manifest in human populations indicates that they are evolutionarily stable (26, 27).

In 1973, John Maynard Smith and George R. Price first introduced game theory from mathematics and economics to the study of animal behavior. Here, the example of an evolutionary game is based on the Hawk-Dove game introduced in Richard Dawkins's The Selfish Gene (28). Organisms do their best to survive and leave many copies of their genes in the gene pool. The hawk's strategy, or phenotype, is to always fight hard and not give in unless it is seriously injured or dead. The dove's strategy is only to threaten and not hurt anyone. When a hawk and a dove fight, the dove runs away and does not suffer any damage. If a hawk meets a hawk, they will fight until one of them is seriously injured or dead. If a dove meets a dove, they will waste time threatening each other until one decides to back down. If players have memories of their opponents, the game cannot be played because they are trying to meet an advantageous opponent. According to Dawkins's calculations, +50 points are assigned for a win, 0 points for a loss, −100 points for injury or death, and −10 points for wasting time. The outcome of any interaction can be scored below.


$$\begin{array}{l} Payoff_{Hawk,Dove}=\begin{array}{ccc} Player & Hawk & Dove\\ Hawk & -25 & +50\\ Dove & 0 & +15 \end{array} \end{array}$$

In a population of doves only, each individual wins half and loses half on average; doves never get hurt or die, but only take threatening positions and waste time. The winner gets +50 – 10 = +40 and the loser gets 0–10 = −10, so the average score is (+40 – 10)/2 = +15. If a hawk invades a population of doves, it always wins, so it has an average score of +50. We can think of the payoff values as equivalent to the fitness (i.e., the reproductive success) of players, in that the winner is the survivor and its genes will spread within the population. A hawk in a population of doves is a highly successful strategy. If a hawk meets a hawk, the average score is (+50 – 100)/2 = −50/2 = −25. If a dove finds itself in a population of hawks, the score is 0, because the dove always loses. However, because it has a score higher than the hawks, the dove gene will spread within the population. A hawk in a group of doves or vice versa is extreme. In a population of hawks and doves, there is a stable proportion of hawks to doves. This happens when the average score of a dove is exactly the same as that of a hawk. The proportion of doves, p is 5/12, the proportion of hawks, 1-p is 7/12, and the average score is +6.25.


$$\begin{array}{l} Expected\;payoff\;value\;of\;the\;hawk\;=-25\left(1-p\right)\;+50p\\ \;Expected\;payoff\;value\;of\;the\;dove\;=0\left(1-p\right)\;+15p \end{array}$$

If all individuals in the population agree to adopt a dove strategy, it is good for the group (+15 vs. +6.25). However, if there is a traitor or a mutant who adopts a hawk strategy, it can increase its average score considerably (+50 vs. +15). It would not be long before the entire population is made of hawks and the average score was back to −25. Again, it is likely that a dove will appear in a population of hawks, and the population will gradually reach a stable ratio of hawks to doves. Organisms with different strategies in a population form, what is called, an evolutionarily stable state, which is the proportion (i.e., the number of doves to hawks) that is the most resistant to mutation within the population.

Here we are looking for a stable ratio of migraineurs to non-migraineurs, i.e., evolutionarily stable. The Hawk-Dove game is a game in which players compete for resources. First, it was assumed that there is reciprocal altruism between migraineurs and non-migraineurs and that they cooperate with each other in dangerous situations. Next, if migraineurs and non-migraineurs in the population compete with each other for cooperation, the non-cooperative Hawk-Dove game can apply to the game between migraineurs and non-migraineurs.

Let us see if the payoff pattern in the Hawk–Dove game is seen in the Migraineur-Non-Migraineur game.

**Figure 2:**
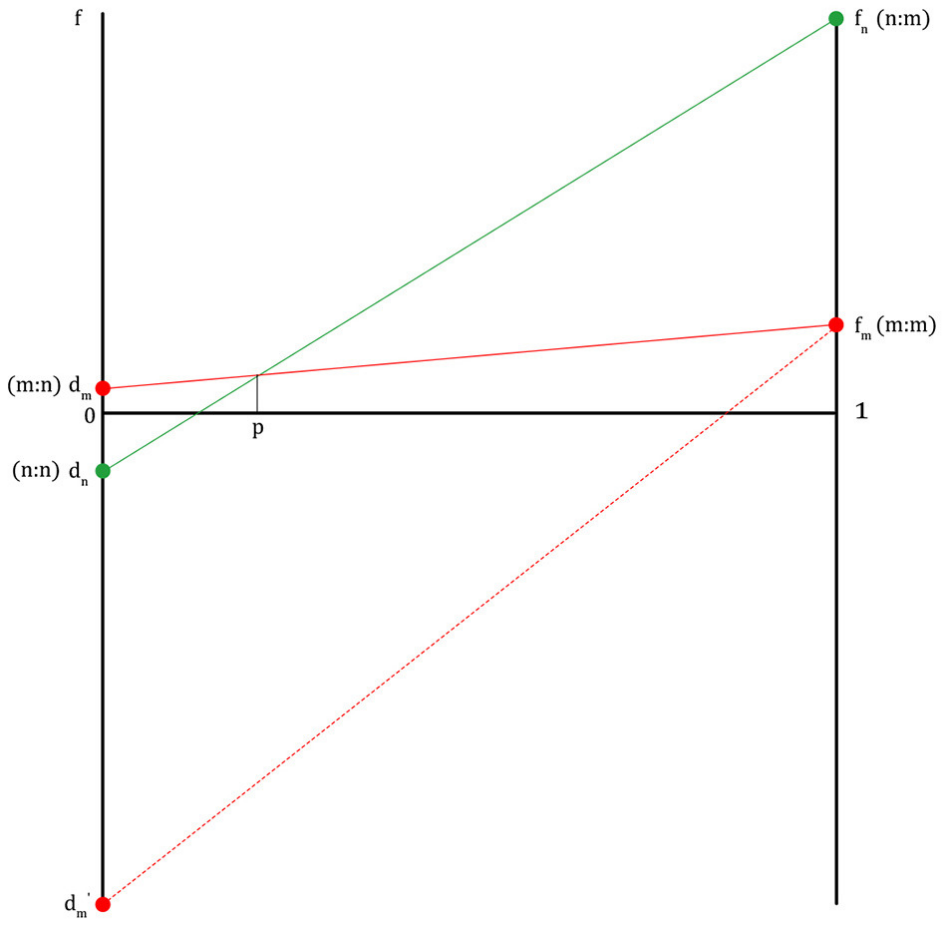
The fitness lines of migraineurs and non-migraineurs on the condition that an agreement between migraineurs and non-migraineurs to help each other in danger is established (m, migraineur; n, non-migraineur; f, fitness; f_m_, fitness of m when m meets m; f_n_, fitness of n when n meets m; d_m_, fitness of m when m meets n; d_n_, fitness of n when n meets n; p, population proportion of migraineurs, at the right end of the graph, 1 is all migraineurs, at the left end, 0 is all non-migraineurs, d_m_′, fitness of m when m is betrayed by n).

**Figure 3:**
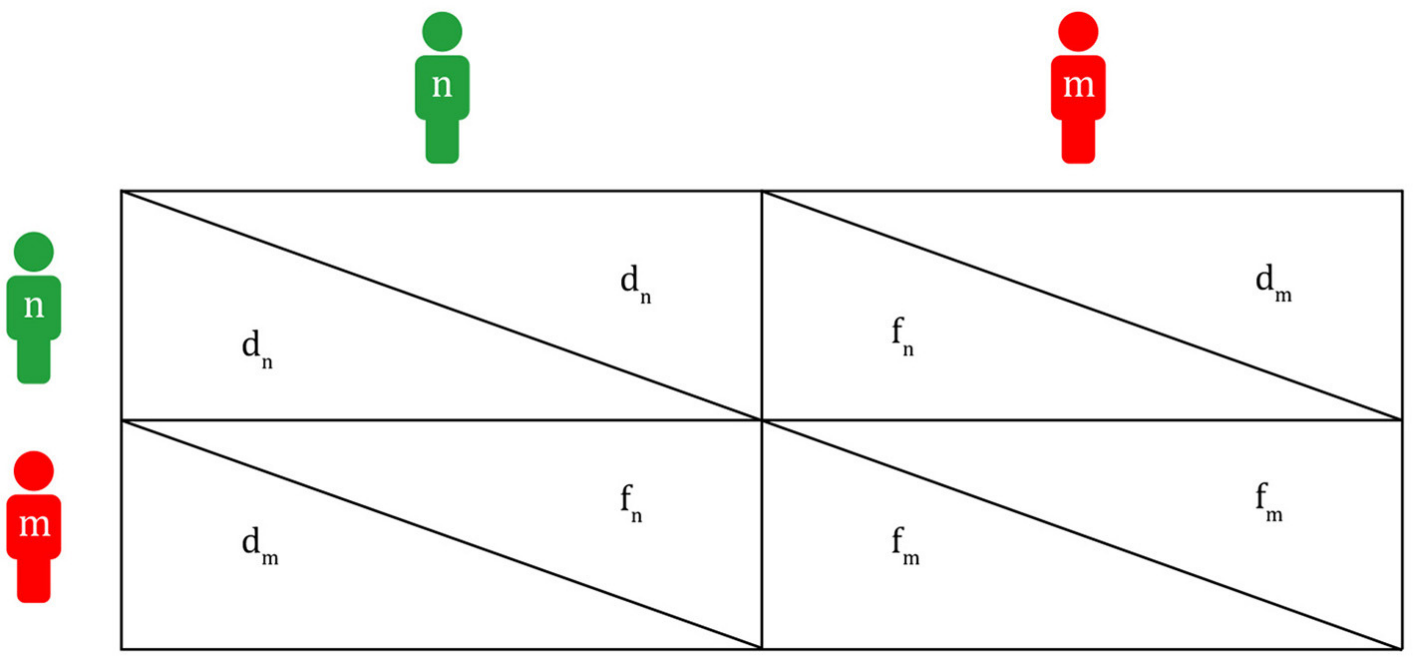
Payoff matrix between a migraineur and a non-migraineur (m, migraineur; n, non-migraineur; f_m_, fitness of m when m meets m; f_n_, fitness of n when n meets m; d_m_, fitness of m when m meets n; d_n_, fitness of n when n meets n).

In Figure 2, f_n_ is the fitness of a non-migraineur that can be obtained by receiving help from a migraineur in times of danger. The f_m_ value is the fitness where each migraineur notices danger and runs away when a migraineur meets a migraineur. The f_m_ value is lower than f_n_ because a migraineur must cover the cost of maintaining a highly sensitive nervous system on normal days. The d_m_ value is the fitness of a migraineur in which the migraineur, in return for helping the non-migraineur in times of danger in the past, receives help from the non-migraineur based on the agreement to help each other in times of danger (d_m_ ≥ 1, if d_m_ is less than 1, there is no reason for the migraineur to help the non-migraineur). d_n_ is the fitness of a non-migraineur, in which a non-migraineur meets a non-migraineur, and each gets through dangerous situations. This pattern, in which the fitness graphs cross each other (f_n>_f_m>_d_m>_d_n_), is like the fitness pattern in the Hawk–Dove game. If this pattern is expressed as a payoff matrix, Figure 3 is obtained.

If there is no agreement between a migraineur and a non-migraineur to help each other in times of danger, the fitness of the non-migraineur is always d_n_ in that the non-migraineur is in danger because they cannot get any information about external threats from the migraineur, and the fitness of the migraineur is always d_m_' in that they cannot get any help from the non-migraineur during a migraine attack. Therefore, only when there is agreement can there be the four fitness values described above, like the pattern in the Hawk–Dove game.

Now, let us combine reciprocal altruism with the game between a migraineur and a non-migraineur.

**Figure 4:**
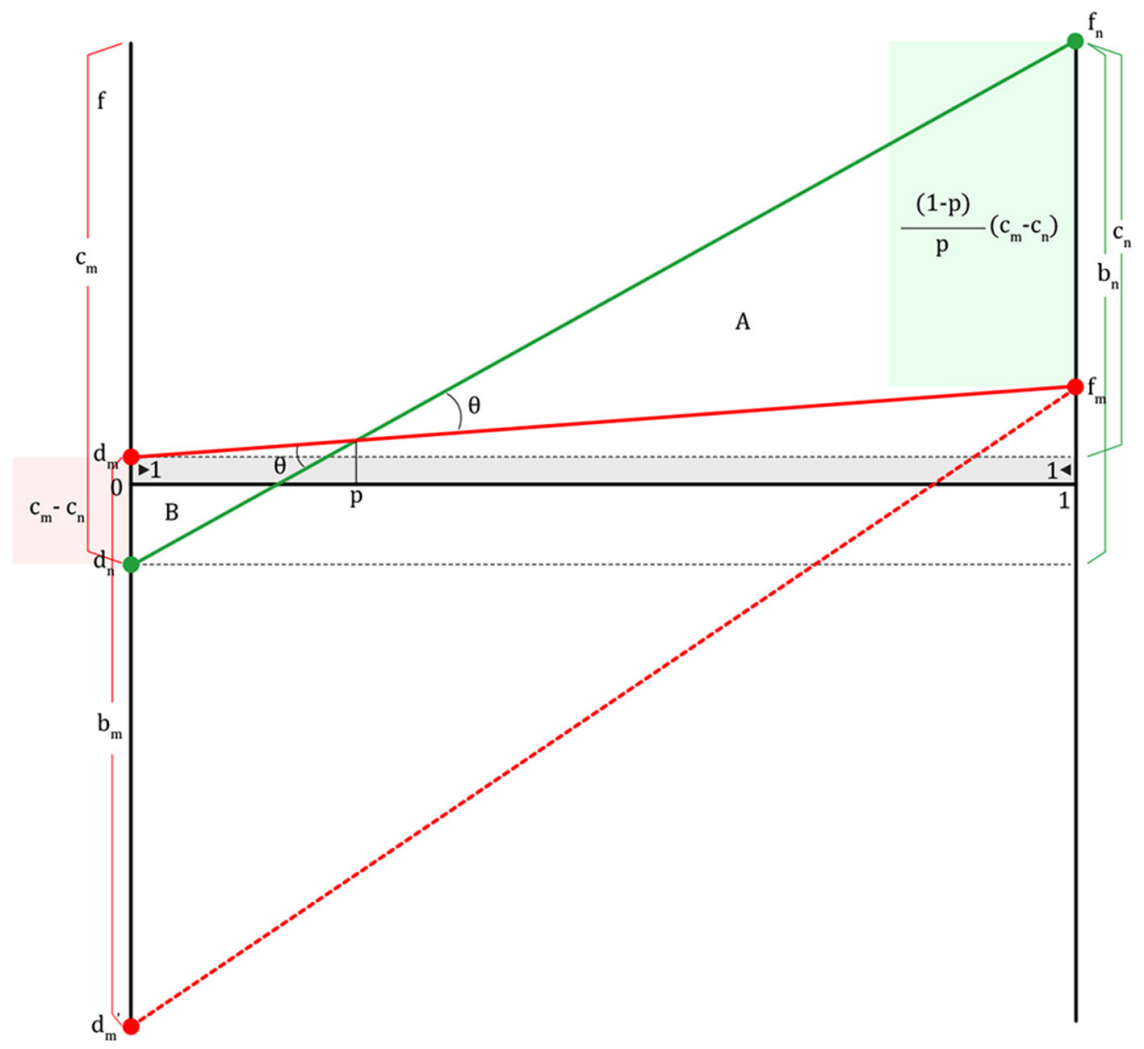
The combination of reciprocal altruism and the game between a migraineur and a non-migraineur (c_m_, cost of migraineur; b_m_, benefit of migraineur; c_n_, cost of non-migraineur; b_n_, benefit of non-migraineur; d_m_, fitness of migraineur when a migraineur meets a non-migraineur; d_m_≥1, p, population proportion of migraineurs).

In Figure 4, c_n_ is the cost of the non-migraineur (f_n_-d_m_) in helping the migraineur escape from danger in return for the migraineur's previous help. The b_n_ value refers to the benefit the non-migraineur (f_n_-d_n_) obtained, if not betrayed, from the migraineur when the non-migraineur was in danger. The c_m_ value is the sum (f_n_-d_n_) of the cost of maintaining the highly sensitive nervous systems on normal days, plus the cost of migraine attacks and the cost of informing the non-migraineur of danger so that they do not get into d_n_. The b_m_ value ranges from d_m_ where the migraineur, in return for helping the non-migraineur in danger in the past, receives help from the non-migraineur to d_m_' where the migraineur is betrayed by the non-migraineur and is seriously injured or dead (d_m_-d_m_′). The d_m_′ value refers to the worst-case scenario in which the cooperation agreement between the migraineur and the non-migraineur is broken. Thinking about only their fitness, it is best for migraineurs to detect danger and escape from danger alone (f_m_). However, if a predator appears during a migraine attack (d_m_), it will certainly need the help of a reliable colleague, a non-migraineur. Therefore, from the standpoint of a migraineur, they need safety insurance.

Now, let us get the values of the payoff matrix between migraineurs and non-migraineurs.

The red-colored and green-colored fitness lines formed by migraineurs and non-migraineurs have the same angle θ, and form two triangles, A and B, are formed, and three sides of each triangle are proportional to each other.

The vertical lines of each of the two triangles, A and B, are also proportional; therefore, the length from f_n_ to f_m_, is $\frac{\left(1-p\right)}{p}\left(c_{m}-c_{n}\right)\;$(Figure 4).

**Figure 5:**
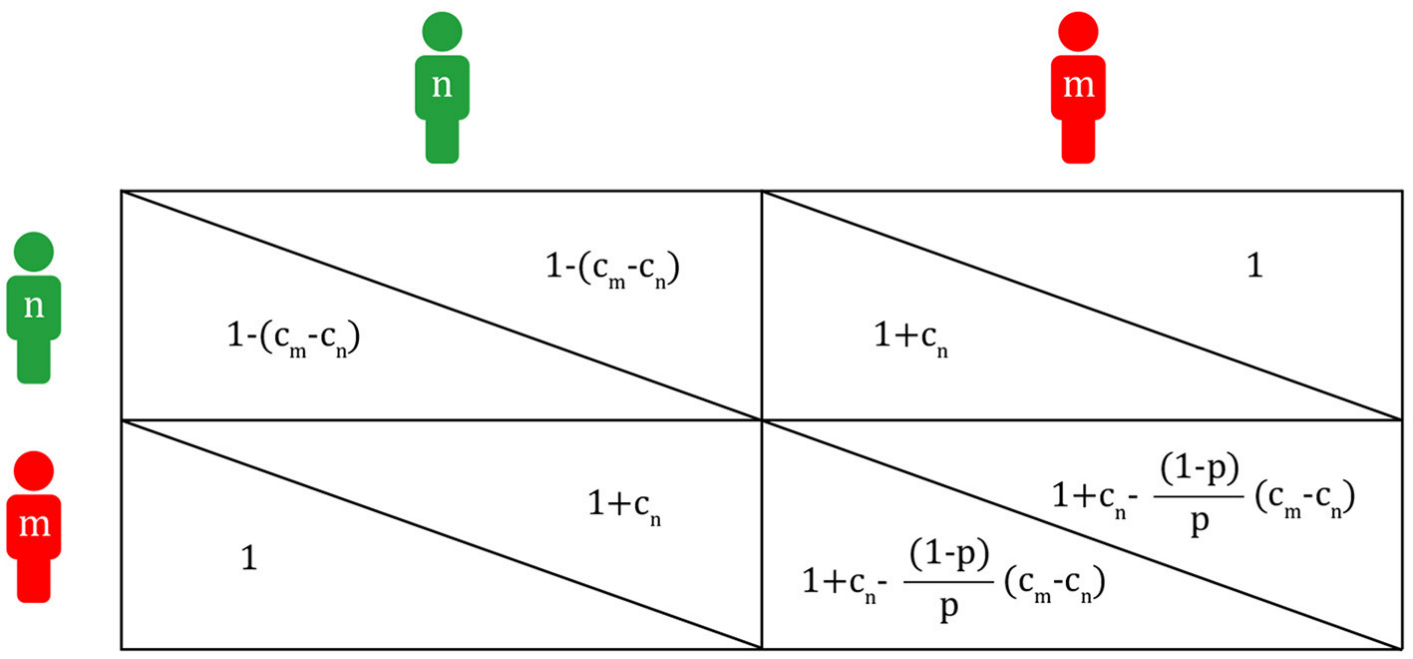
Payoff matrix of the Migraineur-Non-Migraineur game (n, non-migraineur; m, migraineur; c_m_, cost of migraineur; c_n_, cost of non-migraineur; p, population proportion of migraineurs).

Thus, each value of the payoff matrix in Figure 3 may be converted to that in Figure 5.

Each expected value within a group of migraineurs and non-migraineurs is determined by its proportion within the group. If the proportion of migraineurs is p, then the expected payoffs of migraineurs (EV_m_) and non-migraineurs (EV_n_) are,


$$\begin{array}{l} EV_{\text{m}}=(1+c_{n}-\frac{\left(1-p\right)}{p}\left(c_{m}-c_{n}\right))p+1(1-p)\\ \;=1-c_{m}(1-p)+c_{n}\\ \;EV_{\text{n}}=((1-\left(c_{m}-c_{n}\right))(1-p)+(1+c_{n})p\\ \;=1-c_{m}(1-p)+c_{n} \end{array}$$

Here, EV_m_ and EV_n_ are bound to be the same, as the payoff values consisting of c_m_ and c_n_ are obtained, starting from the fixed *p*-value. The point at which the expected values of each other become equal, that is, the vertex where the two triangles A and B meet, can be said to be evolutionarily stable in the population of migraineurs and non-migraineurs. In the Hawk-Dove game, the payoff values are given first, and then p of the evolutionarily stable state is obtained. But, in the Migraineur-Non-Migraineur game, p and c_m_-c_n_ are given first, and each of the payoff values is calculated later. On the right of Figure 4, a non-migraineur in a population of migraineurs is likely to receive help from migraineurs, so the payoff value of the non-migraineur is high, and if a migraineur decides to help the non-migraineur, other migraineurs do not need to help, so the payoff values of migraineurs are also high. However, if migraineurs gradually want to be non-migraineurs for higher payoffs, it is likely that non-migraineurs will meet non-migraineurs, and their payoff values will decrease to d_n_ because they cannot help each other. On the left of Figure 4, a migraineur in a population of non-migraineurs is likely to get help from non-migraineurs, resulting in a good payoff value. Meanwhile, it is probable that non-migraineurs will meet non-migraineurs, getting the worst payoff. Again, if more and more non-migraineurs want to be migraineurs for higher payoff values, they are more likely to meet migraineurs, which increases the possibility of not receiving help in migraine attacks. Eventually, migraineurs and non-migraineurs avoid their worst payoff values, reaching the evolutionarily stable state where each of their payoffs is the same 1-c_m_(1-p) + c_n_.

## 3. Discussion

According to Trivers, reciprocal altruism is altruism that occurs between unrelated individuals when there is a repayment (or at least a promise of repayment) of the altruistic act in the future (25). In order to satisfy reciprocal altruism, individuals must be able to meet repeatedly to exchange help, recognize the individual who helped them, and remember what they helped with. If reciprocal altruism has evolved through natural selection, it should be commonly observed in non-human species in addition to humans. The second and third conditions of reciprocal altruism are based on some intellectual ability, but the first is not. Thus, it seems that genuine examples of reciprocal altruism in non-human species are few and far between (29, 30). Reciprocal altruism has been used to explain the behavioral interaction between migraineurs with a more sensitive sensory nervous system and non-migraineurs with a less sensitive sensory nervous system from the gene-centered view, not the intellectual ability view. However, due to the conditions of reciprocal altruism described above, it is difficult to think that reciprocal altruism between migraineurs and non-migraineurs is purely gene-centered.

Originally, game theory was based on rational decision-making between players. Individual players make decisions, and the payoff to each player depends on the decisions made by all. In nature, organisms compete for resources within a population and interact with each other using certain behavioral strategies. If the strategy for any one organism depends not only on its own strategy but also on the strategies of the other organisms involved, it can also be considered a game. The Hawk-Dove game is a non-cooperative game that competes for resources. However, the Hawk-Dove game has been used to describe an evolutionarily stable state within the population of migraineurs and non-migraineurs; nevertheless, the Hawk-Dove game is a non-cooperative game. To solve this problem, the Hawk-Dove game framework was applied to the game between migraineurs and non-migraineurs on the condition that they compete with each other for cooperation. In the Hawk-Dove game, players do not know the opponent's strategy, but in the game between migraineurs and non-migraineurs, players compete with each other to get more cooperation, in accordance with their pre-arranged agreement. Now, the Migraineur-Non-Migraineur game changes from non-cooperation to cooperation. The game works, and then migraineurs and non-migraineurs, who receive higher payoffs, reproduce at higher rates. Consequently, their genes spread throughout the population. Through these successful strategies, the population ratio of migraineurs to non-migraineurs would reach the evolutionarily stable state, highly resistant to mutation.

Game theory uses rationally determined strategies. On the other hand, the evolutionary game theory uses genetically determined strategies, or phenotypes, that are observable characteristics of organisms. Even though the Migraineur-Non-Migraineur game is transformed into a game of cooperation, cooperation in itself is not entirely a genetically determined strategy because recognizing resources as cooperation requires intellectual ability. Thus, the presence of intellectual ability acting on this game could limit its applicability to an evolutionary game theory. However, if only intellectual ability and not genes were involved in the game, it would not explain why migraines persist in the present environment where dangerous predators have disappeared.

Here, the evolutionary mechanism of migraines was modeled on human brain hypersensitivity. For that, the behavior, or phenotype, of migraineurs must be so powerful as to induce help from non-migraineurs. Migraineurs should be far more sensitive than non-migraineurs so that external threats can be recognized easily. In addition, migraineurs should be found without difficulty within the population. If migraineurs lie down and groan in severe pain during migraine attacks and avoid group activities such as hunting and gathering, they will easily stand out.

**Figure 6:**
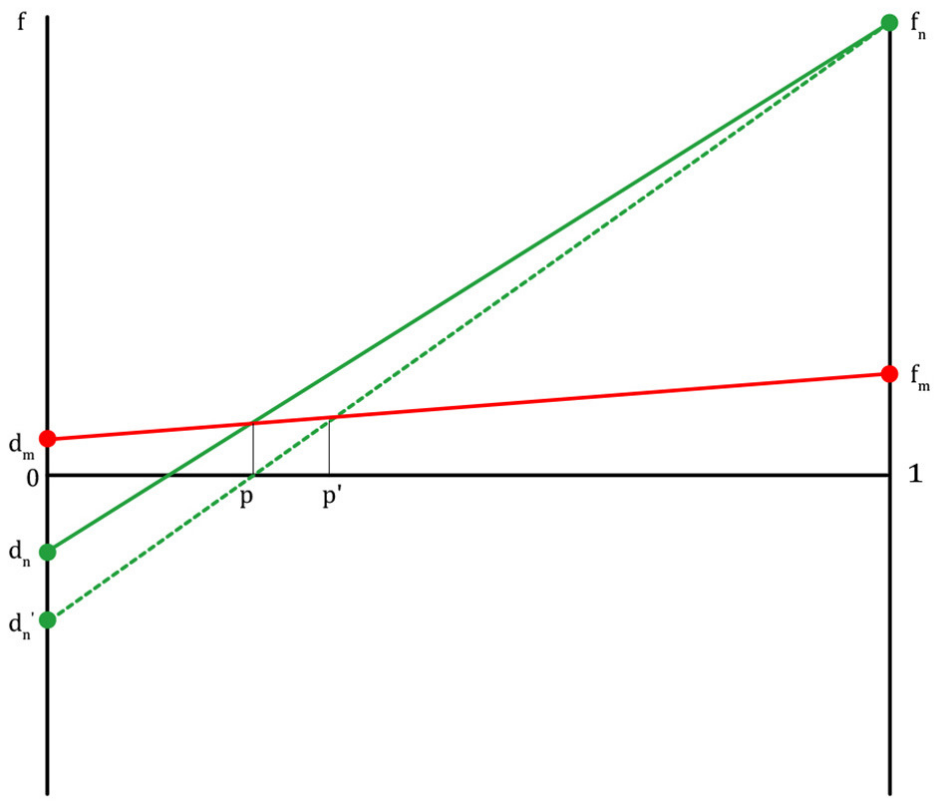
The fitness lines of migraineurs and non-migraineurs: if d_n_ is in a decreasing situation (d_n_ → *d*_n_′), m is increasing (p → *p*′).

There are several things that can be predicted by this game model. If there are more children with poor physical or intellectual ability, or women of childbearing age with poor physical ability in the population (d_n_ → *d*_n_′: if a non-migraineur does not get help from a migraineur, the non-migraineur loses more fitness), the proportion of migraineurs will increase, because they are more likely to be seriously injured or killed by predator attacks (Figure 6). Conversely, if there are more men or adults with good physical or intellectual ability in the population, the proportion of migraineurs will decrease (non-migraineurs win more fitness).

People in high latitudes might get many migraines as a result of over-compensatory reinforcement of other senses instead of being less sensitive to the cold. If they are sensitive to cold and stay indoors instead of going out, this is definitely not going to help their survival and reproduction (31, 32). However, not all migraines are caused by a genetic variant in cold sensitivity. Other factors involved in influencing the prevalence of migraines should be considered (33, 34). In an environment where sensory information from the outside is inevitably reduced, our sensory nervous system needs to have a higher sensitivity in the detection of predators. As *Homo sapiens* migrated from Africa to higher latitudes, living in less light and among more trees, it was more difficult to spot lurking predators. Thus, their sensory nervous system became more sensitive. For this reason, the prevalence of migraine seems to be increasing in Northern Europe (Figure 6).

Our brains have a tendency to pursue hypersensitivity by any means necessary for the earlier detection of advanced threats. Coffee is a favorite food, even though it contains bitter substances. Bitter taste has been regarded as a toxin throughout human evolutionary history. Our belief is not that we have come to like the bitter taste of coffee; what we like is the caffeine in coffee, which stimulates our brains very effectively (35, 36). In the past environment in which our ancestors evolved, the stimulants in food, perhaps, may have helped to keep their brains alert. However, drinking large quantities of coffee increases the risk of migraine attacks because it overexcites our brains (37–39). In addition, a strong preference for sour or spicy foods and cola seems to be due to our brains being addicted to stimulants. Protein is a very important component of our body's growth. This is how our body has created and craved the powerful fifth sense of taste, umami. Of course, fermented fish and cured meats are good sources of protein; however, there is a special reason why only humans love them. They are high in glutamates (40). As a central nervous system stimulant, glutamate can also cause migraines when consumed in large quantities.

As a prerequisite for this evolutionary model, why does the human brain develop hypersensitivity?

First, the cause can be found in the past environment in which humans evolved.

Equatorial Africa, which was covered in tropical rainforest 8–10 million years ago, was split by the Earth's tectonic plate activity. This led to the natural creation of a rift valley, 900–2,700 m high and 50 km wide. The western regions of the rift valley became wet due to rain clouds trapped in the high mountain ranges created by the rift, while the rift valley became hot and dry. This is the East African Rift, which is approximately 4,000 km long from the southern tip of the Red Sea and runs along eastern Africa to eastern Mozambique. Approximately 7 million years ago, the human and chimpanzee lineages separated. *H. sapiens* first appeared in the East African Rift approximately 300,000 years ago and spread to Africa, Europe, and Asia (41). Evolving in the vast open grasslands of the Rift, *H. sapiens* were easily exposed to predators. In this environment, our ancestors, who could quickly and accurately detect predators using their visual, auditory, and olfactory senses, would have achieved high reproductive success. Let us assume that one of our ancestors ran away from a perceived threat thinking it was a lion, but it was not, or that another ancestor carelessly failed to spot a lion and ended up being its meal. The latter is a much greater loss than the former. Therefore, our sensory systems are biased to react sensitively, assuming that there is a predator as soon as a situation is ambiguous.

The second reason can be found in the disproportionate growth of the human brain.

*H. sapiens*, who left the fruit-rich rainforest for the vast open grasslands, should have had a more sensitive vision and hearing to detect far away predators, and a more sensitive sense of smell and taste to identify toxins and rotten meat than chimpanzees, our closest relatives. The brain capacity of early hominins was similar to that of chimpanzees but increased 3 fold in 7 million years. Currently, *H. sapiens* have an average brain capacity of 1,350 cc, and chimpanzees have ˜400 cc. However, looking at the details, only the size of the associative cortices has increased; the sensory cortices have remained the same (42–44). Among the associative cortices in *H. sapiens*, the expansion of the prefrontal cortex is striking. Furthermore, *H. sapiens* have smaller sensory nuclei than other primates (45–48). The size of the olfactory bulb is smaller than that of chimpanzees and other primates, and its structure is simpler. The lamination of the dorsal cochlear nucleus is structurally simple compared to that of prosimians and monkeys. Consequently, they should be as sensitive as possible to overcome simplified sensory nuclei and disproportionately smaller sensory cortices. For example, the primary visual cortex of a migraineur is highly sensitive to fine contrasts and differences present in the visual field (49). This would have been useful in capturing the movements of dangerous predators and prey in the past when our ancestors were still living as hunter-gatherers.

The third reason can be found in the long developmental period of *H. sapiens*.

As *H. sapiens* chose an upright posture, the pelvis decreased in size, and fetuses had to pass through the birth canal before the brains grew larger. Most mammals can stand up and run shortly after birth; humans, however, need adult care for a considerable length of time from birth to walking. For human newborns to achieve brain development comparable to that of chimpanzees, the gestation period must be extended to 18–21 months rather than 9 months (50). This means that *H. sapiens* have a physiological preterm birth. After birth, humans show a slow growth pattern, and even after sexual maturity is achieved, humans still retain the appearance of a child. This is called neoteny (the retention of ancestral juvenile traits in descendant adults of a lineage). For decades, scientists have suggested that mature humans resemble baby chimpanzees and gorillas. Humans have small chins, flat faces, and barely visible hair, stand upright like fetal chimpanzees in their mother's womb and have high-fat content (51–54). Recent studies comparing human and ape brain development support the idea that human brains develop slower than chimpanzees (55). The main difference is that in chimpanzees, the expression of genes that aid in synapse formation peaks at less than 1 year of age, whereas in humans the peak expression extends up to 5 years after birth (56). Furthermore, in chimpanzees, myelination ends by the age of 10 years, whereas in humans it lasts until the age of 30 years (57). Furthermore, the expansion of the associative cortex in humans is related to the slower expression of genes involved in its development and especially that of the frontal cortex. As gene expression slows down, the time it takes the associative cortex to grow increases (58). Therefore, human brain development is significantly delayed. It is also closely related to a unique feature of *H. sapiens*, the prolonged childhood and the onset of adolescence (59). Compared to other primate species, our gestational and infancy periods have been shortened, while our childhoods have been prolonged, and adolescence has been newly added to our developmental periods prior to full maturity. Childhood is a developmental period of participating in various cultural activities and learning adult tasks and skills, during which our bodies are still small. However, our brains reach almost adult size. Adolescence starts with sexual maturity, and a rapid growth spurt occurs. During this time, *H. sapiens*, as apprentices, not only hone the most complex and difficult of adult skills and knowledge but also build friendships with others and look for mates.

Because of prolonged childhoods and the additional developmental period called adolescence, humans are physically weak for a considerably long time (59). During these periods, humans depend on feeding and protection from their parents for survival. In addition, intrinsically, being physically weak necessitates the hypervigilance of the sensory nervous system to find predators. Therefore, it is likely that migraines begin in our childhoods and continue until we have the physical and intellectual ability to cope with the environmental risks.

The fourth reason can be found in the neotenous characteristics of *H. sapiens*.

Neoteny is the retention of ancestral juvenile characteristics in the adult descendants of a lineage. Compared to men, women have a smaller skeleton, smoother ligamentous attachments, smaller mastoid processes, more reduced brow ridges, a more forward-tilted head, larger round eyes, narrower joints, less fetal body hair, a smaller body size, a more backward-tilted pelvis, greater longevity, a lower basal metabolic rate, a faster heartbeat, a more prolonged developmental period, larger tear ducts, and a higher-pitched voice (60). These morphological and physiological features suggest that women are physically weaker and more neotenous than men. To overcome these, a high level of sensitivity in the sensory nervous system is inevitably required in women. Consequently, women are two- to three times more likely to have migraines than men (61, 62).

### 3.1. Toward an evolutionary theory of migraine

Our brains frequently enter hypersensitive states to detect early external threats. If hypersensitive states persist, excessive neurogenic inflammation ensues, subsequently stimulating the trigeminal nerves. Stimulation of the trigeminal nerves causes moderate to severe throbbing pain in the head, or headache, with systemic reactions of a parasympathetic surge (loss of muscle tone, decreased reactivity, hypotension, and bradycardia). Headache and systemic reactions are collectively referred to as migraine. Migraineurs' sensory organs and cortices require a high energy supply to maintain hypersensitive states. When this energy requirement is unusually exceeded during a migraine attack, systemic reactions occur to redistribute our body's energy to the brain, which is the energy-priority organ (63). Thus, they are essential for eliminating neurogenic inflammatory mediators accumulated in sensory organs and cortices, thereby preventing neuronal excitotoxicity (10). It is interesting to note that the systemic reactions of migraine resemble tonic immobility, which is observed in most animals in highly dangerous situations (11, 12). Tonic immobility is a phylogenetically ancient defense behavior (13, 14). Why is this defense behavior observed in migraine? The neural structure responsible for tonic immobility is the ventrolateral periaqueductal gray (vlPAG). Stimulation of the vlPAG leads to the transmission of signals to the rostral ventromedial medulla and spinal cord, causing combined reflexive responses (motor quiescence, decreased reactivity, hypotension, and bradycardia) (64). Indeed, these responses are identical to the systemic reactions of migraine (11–14). Our nervous systems regard the inflammatory reactions in sensory organs and cortices during migraine attacks as very dangerous. In summary, migraine is a defense mechanism to protect our brains from exaggerated hypersensitivity, leading to neuronal excitotoxicity. Describing migraines using a philosophical analogy, the noumenon is “human brain hypersensitivity”, the phenomenon is “defense mechanism”, and the epiphenomenon is “migraine”. The human brain hypersensitivity began to easily recognize external threats, but the human brain, becoming so sensitive, triggers a defense mechanism against excitotoxicity and is eventually expressed as a migraine. In biology, the ultimate goal is an evolutionary explanation of life phenomena, so it makes sense to model the evolutionary mechanism of migraine using game theory. The vast amount of pathophysiological data accumulated so far points to the hypersensitivity of the brains of migraineurs (17–20). Hypersensitivity can occur directly in sensory cortices or sensory organs, or indirectly *via* the hypothalamus, thalamus, PAG-RVM, TCC, and LC (65, 66). Today, it is true that many proximate pathophysiological causes of migraines confuse us in our search to find their ultimate cause (15, 16). Furthermore, more pathophysiological causes will be discovered in the future. Since it is difficult to develop medical therapies for all the pathophysiological causes of migraine, it is more desirable to manage risk factors based on an understanding of the evolutionary cause of migraine.

## 4. Conclusion

Until now, from an evolutionary point of view, we have explained why humans have more sensitive brains. Humans evolved from the African savannah, where survival depended on the hypervigilance of the sensory nervous system to detect predators. Compared to chimpanzees, the brain size of *H. sapiens* is three times larger; however, the increase is not proportional for all parts of the brain. The associative cortices increased while the sensory cortices remained the same. For *H. sapiens*, there are slow periods of growth in childhood and adolescence, which are unique to humans, and in which they are more vulnerable to predators as they are in a state of physical and intellectual weakness. Moreover, *H. sapiens* have the developmental characteristics of neoteny. To overcome all this, human brains should be hypersensitive. Based on human brain hypersensitivity, I have modeled an evolutionary mechanism using reciprocal altruism and the Hawk-Dove game in which migraineurs and non-migraineurs compete with each other for cooperation. Clearly, this combined model should improve the understanding of the pathophysiology of migraine. Migraines have no advantages in the current environment. The basic structures and functions of the human brain have not changed since the end of the Pleistocene, approximately 10,000 years ago (67). Therefore, the human brain, including the body, is not adapted to the present environment but the ancestral environment in which *H. sapiens* lived as hunter-gatherers for 99.9% of its evolutionary history. For that reason, even though we live in the twenty-first century, we still suffer from migraines. Migraine was designed by natural selection to solve problems faced by our ancestors in the distant past.

## Data availability statement

The original contributions presented in the study are included in the article/supplementary material, further inquiries can be directed to the corresponding author.

## Author contributions

The author confirms being the sole contributor of this work and has approved it for publication.
